# Confidence of primary care dental practitioners in the assessment, referral and maintenance of dental implants: a cross-sectional survey in Yorkshire

**DOI:** 10.1038/s41405-026-00456-7

**Published:** 2026-07-07

**Authors:** Jaymit Patel, Martin Ramsdale

**Affiliations:** 1 Leeds Teaching Hospitals Trust, Leeds, UK; 2 NHS England, London, UK

**Keywords:** Dental implants, Restorative dentistry

## Abstract

**Introduction:**

Dental implants are becoming more common in the UK, but the confidence of primary care practitioners in assessing, maintaining, and referring patients with implants is uncertain. Many implant patients struggle to access proper dental care. This study examined the confidence of general dental practitioners (GDPs) in the Yorkshire and Humber region regarding the management of implant patients within the NHS.

**Methods:**

An electronic survey was developed in collaboration with dental clinicians and approved by local dental networks. It included a five-point Likert scale assessing confidence in three clinical scenarios related to implants, alongside twelve yes/no questions about maintenance, referral pathways, and recognition of complications. Thirty-five GDPs completed the survey from January to March 2026.

**Results:**

A total of 35 dental practitioners completed the survey out of a potential 310. All respondents were working in primary care settings in Yorkshire and the Humber at the time of data collection. Respondents reported low confidence across all assessment scenarios, particularly for painful sites (mean 2.29) and those with bleeding (mean 2.37), with over 60% scoring 1 or 2 out of 5. The highest confidence was in oral hygiene instruction (62.9%), while confidence in procedural tasks was low, such as replacing Locator inserts (14.3%) and recognizing mechanical complications (e.g., abutment screw fracture at 20.6%). Only 28.6% were confident in appropriate NHS referrals.

**Conclusions:**

There is potential for a significant gap in the confidence of primary care dental practitioners in Yorkshire and the Humber regarding implant care. This requires broader exploration with a wider sample to characterise trends. Nonetheless our findings underscore the need for targeted education, clearer referral pathways, and structured maintenance protocols to enhance GDPs’ ability to provide safe implant aftercare.

## Introduction

The prevalence of dental implants in the United Kingdom has risen substantially over the past two decades. Two percent of adults in England now have at least one dental implant, which is twice the prevalence in 2009 [1]. The proportion of adults with dental implants increases with age; 8% of individuals aged 55 years and over report having a dental implant [2]. Implant-supported restorations are now considered a well-established and evidence-based treatment modality for the replacement of missing teeth, offering advantages in function, aesthetics and the preservation of alveolar bone. This growing burden of implant aftercare presents a practical challenge for general dental practitioners (GDPs). While implant placement is largely confined to specialist and referral settings, ongoing monitoring, maintenance and management of peri-implant complications falls increasingly to the GDP. Peri-implant diseases affect a substantial proportion of implant patients, with estimates suggesting peri-implantitis affects up to 22% of individuals with dental implant rehabilitations [3]. Timely recognition, appropriate maintenance and expedient referral are therefore critical components of implant survivorship.

Despite this clinical imperative, primary care practitioners may lack the confidence, training and clearly defined referral frameworks required to manage these patients effectively. A number of studies have explored GDP awareness of peri-implant disease [4–6]. However, contemporary evidence focused on commissioning the National Health Service (NHS) services and pathways is limited. Dental access in the United Kingdom can be provided by NHS or private dental practices, with some practices offering a mix of both. NHS services are commissioned regionally and are supported to treat the patient population through contract management and the delivery of training by Workforce Training and Education (WTE). Research exploring the confidence level of dentists working for the NHS to assess and treat patients with dental implants is lacking.

This study was designed to address that gap. The survey was co-developed through structured discussions with practising dental clinicians and was approved by the South Yorkshire, West Yorkshire and Humber North Yorkshire Local Dental Networks (LDNs), and the Managed Clinical Network (MCN) for Restorative Dentistry in Yorkshire and the Humber. The survey data will be used to guide pathway and skillset development. The objectives were to (1): quantify GDP confidence in the clinical assessment of implant sites presenting in different states; (2) assess confidence in performing a range of implant maintenance procedures; and (3) evaluate confidence in referral pathways for implant-related problems within NHS settings.

## Methods

### Study design

This study employed a cross-sectional survey design. An anonymous, self-administered electronic questionnaire was distributed to dental practitioners working in primary care across the Yorkshire and the Humber region. The survey was conducted between January and March 2026.

### Survey development and ethical approval

The questionnaire was co-produced through iterative consultation with local dental practitioners and refined following discussion at meetings of the Yorkshire and the Humber Local Dental Networks (LDNs). The instrument was pilot tested in the first instance to assess completion time, comprehension, and to address any queries. Volunteer dental practitioners from the stakeholder group were utilised for this process. Feedback was collected via email, and a follow-up meeting was held to discuss it. The final instrument was reviewed and approved by the Managed Clinical Network for Restorative Dentistry in Yorkshire and the Humber. All responses were fully anonymised at the point of data collection. As an anonymised service evaluation approved through established network governance processes, formal NHS Research Ethics Committee approval was not required; however, the study was conducted in accordance with the Declaration of Helsinki and Good Clinical Practice principles. Furthermore, as this was a voluntary survey undertaken by dental professionals, completing the survey implied consent. The survey was distributed via Local Dental Networks (LDNs), Local Dental Councils (LDCs), the Yorkshire and the Humber ICBs, and the Restorative Dentistry Managed Clinical Network (MCN).

Any general dental practitioner working in the primary care setting and offering NHS dental services was considered eligible. All NHS GDPs in the region were eligible to take part. As no formal power calculation was carried out, descriptive statistics only were reported. The survey was distributed via email and was completed using Microsoft Forms within an NHS account. Eligibility requirements were included in the email, and were also included in the survey instrument.

### Survey instrument

The questionnaire comprised two sections. The first assessed respondent confidence in the clinical assessment of three implant site presentations using a five-point Likert scale (1 = lowest confidence, 5 = highest confidence): (i) an asymptomatic implant site; (ii) a painful implant site; and (iii) an implant site exhibiting bleeding on probing and suppuration.

The second section comprised twelve binary (yes/no) questions assessing respondent confidence in: (a) five maintenance and procedural tasks (oral hygiene instruction for implant sites, non-surgical debridement, cement removal, re-restoration of screw access restorations, and replacement of Locator inserts); (b) referral to appropriate private and NHS services; and (c) recognition of five clinical complications (peri-implant mucositis, peri-implantitis, screw loosening, abutment screw fracture, and implant fracture) [7].

### Data analysis

Descriptive statistics were used to analyse all responses. For Likert-scale items, means, standard deviations (SDs) and medians were calculated. Score distributions were tabulated, with groupings into low confidence (scores 1–2) and high confidence (scores 4–5) reported as proportions. For binary items, frequencies and percentages of ‘yes’ responses were calculated. Analyses were performed using Microsoft Excel. No inferential statistical testing was conducted given the exploratory nature of this study.

## Results

### Respondent characteristics

A total of 35 dental practitioners completed the survey out of a potential 310. All respondents were working in primary care settings in Yorkshire and the Humber at the time of data collection. Demographic data (including qualification type, year of graduation and practice setting) were not collected in this survey iteration in order to minimise barriers to participation and preserve full anonymity.

### Confidence in clinical assessment

Overall, respondents demonstrated low to moderate confidence in the assessment of implant sites across all three clinical scenarios (Table 1). The highest mean confidence score was recorded for the assessment of asymptomatic implant sites (mean 2.71, SD 1.34, median 3.0), although 42.9% of respondents (*n* = 15) scored this item at 1 or 2, indicating low confidence. Only 28.6% (*n* = 10) rated their confidence at 4 or 5.

**Table 1:** Confidence in clinical assessment of dental implant sites (five-point Likert scale; 1 = lowest, 5 = highest confidence; *n* = 35) SD standard deviation.

Assessment scenario	*n*	Mean (SD)	Median	Score 1–2 (low confidence)	Score 3 (neutral)	Score 4–5 (high confidence)
Asymptomatic implant site	35	2.71 (1.34)	3	15 (42.9%)	10 (28.6%)	10 (28.6%)
Painful implant site	35	2.29 (1.23)	2	21 (60.0%)	8 (22.9%)	6 (17.1%)
Implant site with bleeding and suppuration	35	2.37 (1.19)	2	22 (62.9%)	6 (17.1%)	7 (20.0%)

Confidence was considerably lower when assessing painful implant sites (mean 2.29, SD 1.23, median 2.0), with 60.0% (*n* = 21) of respondents scoring 1 or 2, and only 17.1% (*n* = 6) indicating high confidence. Similarly, assessment of an implant site with bleeding on probing and suppuration produced a mean score of 2.37 (SD 1.19, median 2.0), with 62.9% (*n *= 22) reporting low confidence and only 20.0% (*n *= 7) reporting high confidence.

### Confidence in maintenance tasks

Oral hygiene instruction was the maintenance task in which respondents demonstrated greatest confidence, with 22 of 35 (62.9%) responding positively. However, confidence in more procedural tasks was markedly lower. Non-surgical debridement of implant sites was performed with confidence by only 13 respondents (37.1%), while cement removal was accessible to 11 (31.4%). Re-restoration of screw access restorations was reported confidently by only 9 respondents (25.7%), and replacement of Locator inserts by just 5 (14.3%), representing the lowest confidence across the entire survey (Table 2).

**Table 2:** Respondent confidence in maintenance tasks, referral pathways and recognition of complications (binary yes/no responses) Values represent number (*n*) and percentage (%) of respondents answering ‘Yes’ to confidence in each item.

Task / Competency	*n*	Yes *n* (%)	No *n* (%)
**Maintenance tasks**			
Oral hygiene instruction for implant sites	35	22 (62.9%)	13 (37.1%)
Non-surgical debridement of implant sites	35	13 (37.1%)	22 (62.9%)
Cement removal	35	11 (31.4%)	24 (68.6%)
Re-restoration of screw access restorations	35	9 (25.7%)	26 (74.3%)
Replacement of Locator inserts	35	5 (14.3%)	30 (85.7%)
**Referral**			
Appropriate referral to private services	35	19 (54.3%)	16 (45.7%)
Appropriate referral to NHS services	35	10 (28.6%)	25 (71.4%)
**Recognition of complications**			
Peri-implant mucositis	35	15 (42.9%)	20 (57.1%)
Peri-implantitis	35	17 (48.6%)	18 (51.4%)
Screw loosening	34	14 (41.2%)	20 (58.8%)
Abutment screw fracture	34	7 (20.6%)	27 (79.4%)
Implant fracture	35	7 (20.0%)	28 (80.0%)

### Confidence in referral pathways

Confidence in referral was to private services was higher than for NHS pathways. Just over half of respondents (19/35, 54.3%) felt confident in making an appropriate referral to a private implant service, compared with fewer than one third (10/35, 28.6%) who felt confident in identifying the correct NHS referral pathway.

### Recognition of peri-implant diseases and mechanical complications

Recognition of peri-implant diseases was moderate but remained below 50% for both conditions: 48.6% (*n *= 17) of respondents felt confident recognising peri-implantitis and 42.9% (*n* = 15) peri-implant mucositis. Recognition of mechanical complications was substantially lower: screw loosening was recognised confidently by 14 respondents (41.2%), while abutment screw fracture (7/34, 20.6%) and implant fracture (7/35, 20.0%) were recognised by only one in five.

## Discussion

### Principal findings

This survey reveals significant and clinically important gaps in the confidence of primary care dental practitioners in Yorkshire and the Humber with respect to the assessment, maintenance and referral of patients with dental implants. Across all domains examined (assessment, procedural maintenance, referral knowledge and complication recognition) a substantial proportion of respondents reported low or absent confidence. These findings are of direct relevance to patient safety and to the sustainability of NHS implant aftercare.

### Assessment confidence

The finding that fewer than 30% of respondents rated themselves as highly confident in assessing even an asymptomatic implant site is notable, particularly given that routine dental examination is a core GDP competency [8, 9]. Specific reference to implants is made in relation to outcomes within the GDC framework document *The Safe Practitioner: A framework of behaviours and outcomes for dental professional education - Dentist* [9]*; ‘explain the use of implants as a treatment option, including their outcomes, limitations, and risks’*. Whilst other domains in the same document may not specifically reference implants, they would be applicable to the care of patients with implants e.g. ‘*Monitor and review treatment outcomes and patient response to advice, providing aftercare, follow-up and ongoing preventive advice and intervention’* This likely reflects the limited focus on implant assessment within undergraduate dental education, where implant dentistry has historically received less attention than restorative or oral surgery curricula. The steep decline in confidence when assessing symptomatic presentations, with over 60% of respondents reporting low confidence in evaluating sites with bleeding and suppuration, raises important questions about the capacity of primary care to identify and act upon early signs of peri-implant disease.

Peri-implant mucositis, characterised by reversible mucosal inflammation around an implant, is an important risk factor for peri-implantitis, an irreversible destructive condition associated with progressive crestal bone loss [10, 11]. Early identification and intervention at the mucositis stage is critical [12, 13]; the finding that fewer than half of respondents felt confident recognising either condition underscores the potential for disease progression going undetected in primary care settings.

### Maintenance and procedural competency

A clear gradient in confidence was observed across maintenance tasks, ranging from oral hygiene instruction (a familiar component of general dental practice) to highly implant-specific technical procedures. Whilst 62.9% of respondents reported confidence in providing implant-specific oral hygiene instruction, this figure represents a minority of practitioners who are fully prepared for one of the most fundamental components of implant maintenance. The very low confidence in replacing Locator inserts (14.3%) and re-restoring screw access restorations (25.7%) likely reflects both the specialist nature of these tasks and the limited availability of postgraduate training in prosthetic implant maintenance.

Non-surgical debridement of implant sites requires familiarity with implant-compatible instruments (e.g. plastic or titanium scalers, air-polishing devices) to avoid surface damage to implant components. Only 37.1% of respondents felt confident in this task and this may be suggestive that practitioners might potentially use incorrect instrumentation or defer maintenance, neither of which represents an optimal clinical outcome. However, knowledge levels were not assessed as part of the survey so practitioners, may be aware of and/or undertake appropriate maintenance procedures, but lack confidence in the approach.

### Referral pathways

Perhaps the most actionable finding of this survey is the poor understanding of NHS implant referral pathways, with only 28.6% of respondents reporting confidence in making an appropriate NHS referral. This figure is consistent with broader concerns about the clarity and accessibility of NHS restorative dentistry referral routes, particularly post-COVID-19 reconfiguration of dental services. The absence of a clearly signposted, regionally agreed pathway for implant-related referrals is likely to result in delayed access to specialist care, patient frustration, and the risk of complications being managed beyond the competency of the referring practitioner.

Notably, confidence in private referral (54.3%) was almost double that for NHS pathways, reflecting both the predominantly private context in which implant treatment is provided in the UK and the more established direct referral routes that exist in the independent sector. NHS ICB dental commissioners and local clinical networks could consider whether explicit, widely distributed guidance on NHS referral pathways for implant complications could address this knowledge gap.

### Comparison with existing literature

The findings of this study are broadly consistent with published research from other regions [14]. Studies examining GDP knowledge of peri-implant disease have similarly identified low levels of awareness and limited clinical experience [15, 16]. A recurring theme in the literature is the mismatch between the growing number of implant-carrying patients presenting in primary care and the preparedness of the dental workforce to manage them. The Yorkshire and the Humber findings are important because they provide regionally specific, network-validated data to support targeted service development and continuing professional development (CPD) planning.

### Limitations

Several limitations of this study should be acknowledged. The sample size of 35 respondents is low, and whilst consistent with similar survey-based studies in this field, limits the statistical power and generalisability of the findings. The sample size in regional primary care surveys is often limited. Methods were utilised to maximise the response rate, primarily by engaging a wide range of stakeholders. An alternative would involve dissemination via social media and other online resources, which support snowball sampling. The limitation of this approach is, however, that a true response rate is often difficult to calculate. Furthermore, this may risk expanding the survey beyond the target population. An alternative would be to incorporate a similar set of questions into a pre-existing national survey.

The survey did not collect demographic data including year of qualification, postgraduate training background or proportion of patients with implants within each respondent’s clinical caseload. Such data would be valuable in identifying subgroups at greatest need of targeted training and support.

Participants scored self-reported confidence only; without an assessment of knowledge. This limits the external validity and comparability of the study to findings from other regions. We propose that this is investigated through workshopping activities associated with educational sessions, thus also facilitating an assessment of the effectiveness of training sessions.

Respondent self-selection bias is inherent in voluntary survey designs; practitioners with a particular interest in or awareness of implant dentistry may be over-represented. The binary yes/no format of the second section, whilst pragmatic, does not capture nuance in the degree of confidence or uncertainty, and future studies may benefit from applying a consistent Likert scale throughout. Finally, the survey was conducted in a single region of England, and findings may not be representative of the national picture.

### Implications for practice and education

This study provides compelling evidence for several areas of targeted action. Firstly, there is a clear need for accessible, high-quality postgraduate CPD focusing on peri-implant disease recognition and non-surgical maintenance in primary care. Secondly, the wide dissemination of (with accompanying education as required) accepted implant referral protocols.

Thirdly, low confidence in assessing even asymptomatic implant sites suggests that integrating implant examination into routine dental assessment needs to be normalised through both education and the development of clinical checklists or pro forma tools. Instruments such as the peri-implant chart (analogous to the Basic Periodontal Examination (BPE)) could provide a structured framework to support assessment in primary care. Finally, co-production of solutions with the clinical community, as was the approach adopted in developing this survey, is recommended as a model for translating findings into sustainable change.

## Conclusions

Within the limitations of this study, and in particular the low response rate, there is potential for substantial gaps in confidence relating to the assessment, maintenance and referral of patients with dental implants. The majority of respondents reported low confidence in assessing symptomatic implant presentations, recognising peri-implant diseases and identifying appropriate NHS referral pathways. Confidence in implant-specific procedural maintenance tasks was consistently low, particularly for prosthetic interventions. These findings highlight the urgent need for structured postgraduate education, clear and accessible referral frameworks, and greater integration of implant assessment into the routine primary care dental examination. This study, co-developed and approved through Yorkshire and the Humber's network governance structures, provides a foundation for targeted regional action to support the primary care dental workforce in delivering safe and effective long-term implant care.
